# Creatine is a Conditionally Essential Nutrient in Chronic Kidney Disease: A Hypothesis and Narrative Literature Review

**DOI:** 10.3390/nu11051044

**Published:** 2019-05-10

**Authors:** Adrian Post, Dimitrios Tsikas, Stephan J.L. Bakker

**Affiliations:** 1 Department of Internal Medicine, University Medical Center Groningen, University of Groningen, 9713 GZ Groningen, The Netherlands; s.j.l.bakker@umcg.nl; 2 Institute of Toxicology, Core Unit Proteomics, Hannover Medical School, Carl-Neuberg-Str. 1, 30625 Hannover, Germany; Tsikas.Dimitros@mh-hannover.de

**Keywords:** creatine, creatinine, chronic kidney disease, essential nutrient, AGAT

## Abstract

To accommodate the loss of the plethora of functions of the kidneys, patients with chronic kidney disease require many dietary adjustments, including restrictions on the intake of protein, phosphorus, sodium and potassium. Plant-based foods are increasingly recommended as these foods contain smaller amounts of saturated fatty acids, protein and absorbable phosphorus than meat, generate less acid and are rich in fibers, polyunsaturated fatty acids, magnesium and potassium. Unfortunately, these dietary recommendations cannot prevent the occurrence of many symptoms, which typically include fatigue, impaired cognition, myalgia, muscle weakness, and muscle wasting. One threat coming with the recommendation of low-protein diets in patients with non-dialysis-dependent chronic kidney disease (CKD) and with high-protein diets in patients with dialysis-dependent CKD, particularly with current recommendations towards proteins coming from plant-based sources, is that of creatine deficiency. Creatine is an essential contributor in cellular energy homeostasis, yet on a daily basis 1.6–1.7% of the total creatine pool is degraded. As the average omnivorous diet cannot fully compensate for these losses, the endogenous synthesis of creatine is required for continuous replenishment. Endogenous creatine synthesis involves two enzymatic steps, of which the first step is a metabolic function of the kidney facilitated by the enzyme arginine:glycine amidinotransferase (AGAT). Recent findings strongly suggest that the capacity of renal AGAT, and thus endogenous creatine production, progressively decreases with the increasing degree of CKD, to become absent or virtually absent in dialysis patients. We hypothesize that with increasing degree of CKD, creatine coming from meat and dairy in food increasingly becomes an essential nutrient. This phenomenon will likely be present in patients with CKD stages 3, 4 and 5, but will likely be most pronouncedly present in patients with dialysis-dependent CKD, because of the combination of lowest endogenous production of creatine and unopposed losses of creatine into the dialysate. It is likely that these increased demands for dietary creatine are not sufficiently met. The result of which, may be a creatine deficiency with important contributions to the sarcopenia, fatigue, impaired quality of life, impaired cognition, and premature mortality seen in CKD.

## 1. Introduction

More than 10% of the world’s population is currently suffering from non-dialysis-dependent chronic kidney disease (CKD) [1,2], and almost one out of every two adults aged 30–64 years is expected to develop CKD during their lifetime [3,4]. Healthcare expenditures escalate as non-dialysis-dependent CKD progresses and explodes once dialysis becomes necessary, as exemplified by the fact that while patients with dialysis-dependent CKD only make up 0.5% of the U.S. population, costs for their care takes 7% of the total U.S. health budget [4,5,6]. It is recognized that the escalating costs in CKD are largely due to an increasing burden of comorbidities as CKD progresses, with by far the highest burden in patients on dialysis [4,6]. The comorbidities that accompany CKD are for a large part the progressive loss of the plethora of functions displayed by the kidneys, including maintenance of protein homeostasis, acid-base homeostasis, volume homeostasis, and bone and mineral homeostasis [7,8]. This requires many dietary adjustments, including restriction of intake of protein, phosphorus, sodium and potassium. While dietary guidelines endorse a balanced intake of both animal and plant proteins [9,10,11,12,13,14], plant-based foods are increasingly recommended in literature as these foods contain smaller amounts of saturated fatty acids, protein and absorbable phosphorus than meat, generate less acid and are rich in fibers, polyunsaturated fatty acids, magnesium and potassium [8,15]. The National Kidney Foundation website names a plant-based diet as being beneficial to CKD patients [16,17,18]. Unfortunately, these dietary recommendations cannot prevent the occurrence of many symptoms, which typically include fatigability, muscle weakness, muscle wasting, and sarcopenia [7,8,19].

## 2. Compromised Creatine Intake

**Figure 1:**
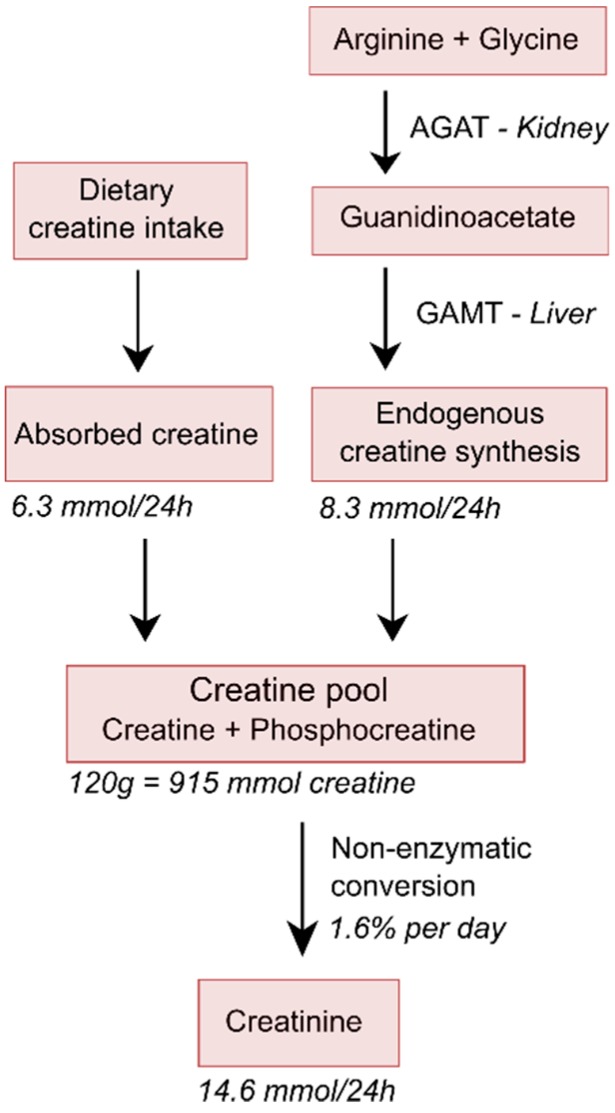
Simplified schematic overview of creatine homeostasis. It has been estimated that young 70 kg-weighing men with normal muscle mass contains about 120 g of total creatine (creatine and phosphocreatine), which equals 915 mmol of creatine, since its molecular weight is 131 g/mol. With a conversion rate of 1.6% per day, and the slightly lower molecular weight of creatinine of 113 g/mol, this will result in a daily 24 h urinary creatinine excretion of approximately 1.65 g, which is equivalent to 14.6 mmol, and thus also equivalent to a loss of 14.6 mmol of creatine, which implies a loss of 1.91 g of creatine per day. The average diet of young omnivorous 70 kg-weighing men has been estimated to contain 7.9 mmol of creatine per day, which with an intestinal absorption rate of 80%, will result in an uptake of creatine from the diet of 6.3 mmol/day, resulting in requirement of endogenous creatine synthesis at a rate of 8.3 mmol/day required to remain in steady state. Because of their lower muscle mass and dietary intake, rates for women would be about 70–80% of that in men [27].

One threat coming with the recommendation of low-protein diets in patients with non-dialysis-dependent CKD and with high-protein diets in patients with dialysis-dependent CKD, particularly with an increasing focus towards proteins coming from plant-based sources, is that of creatine deficiency. However, existing recommendations and guidelines for diets in patients with CKD [7,8,19,20,21] do neither mention creatine intake, nor the potential threat of creatine deficiency. Also, evaluations of risk of development of protein-energy malnutrition with loss of nutritional components into the dialysate do neither mention creatine intake, nor a potential threat of creatine deficiency [22,23,24]. More than 90% of the body’s creatine and phosphocreatine is present in muscle [25], of which in resting muscle cells one-third exists in the form of creatine and two-thirds in the form of phosphocreatine, with concentrations of phosphocreatine reaching up to 30 mmol/L [26]. In tissues with highly variable energy demands, like muscles, this phosphocreatine serves as an energy buffer par excellence [26]. In the field of CKD, creatine is best known for its continuous, low-grade, non-enzymatic degradation to creatinine, which accounts for 1.6–1.7% of the total creatine pool per day [27]. Because the degradation product creatinine is subject to complete glomerular filtration in the kidney, and no tubular reabsorption, plasma creatinine is a well-established renal function marker. To compensate for this daily loss, the creatine pool requires continuous replenishment. For a 70 kg-weighing man with normal muscle mass, the daily loss of creatine as creatinine is estimated to be 14.6 mmol (Figure 1). The average diet of a young omnivorous 70 kg-weighing man has been estimated to contain 7.9 mmol of creatine per day, which with an intestinal absorption rate of 80% will result in an uptake of creatine from the diet of 6.3 mmol/day. Therefore, an endogenous creatine synthesis at a rate of 8.3 mmol/day is required to remain in steady state. Endogenous creatine synthesis involves two enzymatic steps [28]. The first step is a metabolic function of the kidney facilitated by the enzyme arginine:glycine amidinotransferase (AGAT), which converts arginine and glycine into guanidinoacetate. The second step is facilitated by the enzyme guanidinoacetate N-methyltransferase (GAMT) in the liver, which converts guanidinoacetate to creatine by performing a methylation step. Since, in food, creatine is exclusively of animal origin and since muscle meats are the primary sources (dairy products generally supply, at most, 20% of dietary creatine), it is apparent that vegetarians need to synthesize all (vegans) or nearly all (lacto-ovo vegetarians) of their creatine. That maintenance of creatine pools is compromised under these circumstances is notified by the fact that vegetarians have been shown to have lower muscle creatine levels than non-vegetarians [29]. The same is true for levels of creatine in plasma and in red blood cells, conclusively resulting from insufficient compensatory endogenous creatine synthesis [30,31].

## 3. Contribution of the Kidney to Endogenous Synthesis of Creatine in Humans

The regulation of endogenous creatine synthesis occurs at the level of AGAT [32,33,34,35,36], and dietary creatine represses renal AGAT expression and activity, thereby regulating endogenous synthesis of creatine [33,34,35,36]. Although kidneys are the main side of AGAT expression in all mammals, there is considerable variation in extra-renal AGAT expression between species [28]. In humans, highest activities are present in kidney and pancreas, while other tissues, such as brain and testes, express lower activities [27,28]. On the basis of the fact that the rate of creatine biosynthesis is considerably reduced in nephrectomized rats and rabbits [37,38,39], it has long been considered that the main route of endogenous creatine synthesis in mammals involves the formation of guanidinoacetate by AGAT in the kidney [28]. The actual potential to which the kidney contributes to the endogenous synthesis of creatine in circumstances of low or absent intake of creatine in humans is unclear. Because both intake of creatine and dehydration have been shown to downregulate creatine synthesis at the level of AGAT gene expression in the kidney [33,34,35,36], experiments to establish the actual potential of the kidney to contribute to endogenous creatine synthesis should preferably be done under controlled conditions, with assurance of absent or low creatine intake and certification of a sufficient hydration status, to allow for maximal contribution of the kidney to endogenous creatine synthesis. A study in humans, in which the renal production of guanidinoacetate by the kidney was estimated by measuring the difference in guanidinoacetate concentrations between arterialized blood and renal venous blood samples, suggests that the kidney only contributes ~20% to total endogenous guanidinoacetate synthesis [40,41]. It should, however, be noted that measurements to come to this conclusion were performed while participants were not on a creatine-free diet. Also, the protocol that was used for measurements of renal blood flow in the concerned study does neither report on taking into account the incomplete renal extraction of *para*-amino hippuric acid, nor does it report on taking into account that subjects should be in steady-state during measurements, both contributing to a high likelihood of underestimation of true renal blood flow [42,43]. Because subjects used a standard meal prior to measurements, it is highly unlikely that they achieved steady-state during measurements, and the high carbohydrate content of a meal is likely to have adversely influenced renal extraction of *para*-amino hippuric acid [44]. Calculations were also made using renal blood flow rather than renal plasma flow, while it is known that neither creatine nor guanidinoacetate are in free equilibrium with plasma concentrations, making it necessary that calculations are made based on renal plasma flow rather than renal blood flow [41]. Moreover, the investigators used arterialized venous blood rather than arterial blood to estimate arterial concentrations of renal arterial guanidinoacetate concentrations, a method which would have required validation before application, because it may have resulted in an overestimation of arterial concentrations due to the admixture of venous blood [45,46]. The combination of potential suppression of renal AGAT activity to below its maximal achievable level and a high likelihood of underestimation of true endogenous guanidinoacetate synthesis, makes it very likely that the contribution of the kidney to endogenous guanidinoacetate synthesis can be higher in circumstances of low creatine intake. In another study in humans—in which arterial rather than arterialized venous samples were taken—but subjects were also not on a creatine-free diet and in which hydration status was also not certified, increases in venous compared to arterial plasma guanidinoacetate concentrations ranged between 12–47%, consistent with renal guanidinoacetate synthesis taking place in every subject, with great variation between subjects, possibly depending on diet and hydration status [41].

**Figure 2:**
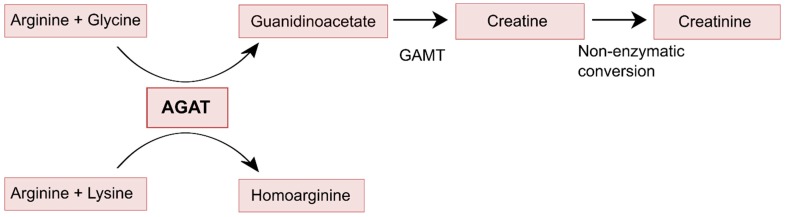
Enzymatic functions of AGAT, showing that guanidinoacetate is further metabolized to creatine and eventually creatinine, while homoarginine is not further metabolized.

Data from studies that we have performed on homoarginine homeostasis in healthy kidney donors and renal transplant recipients suggest that renal contribution to guanidinoacetate synthesis can be even higher if one takes into account that also the arginine required for its synthesis is produced by the kidney [47,48]. For this, it should be realized that the synthesis of homoarginine from arginine is also mediated by the enzyme AGAT, with lysine as a substrate instead of glycine and that, unlike guanidinoacetate—which is metabolized to creatine—homoarginine is currently only known as a metabolic end product, of which urinary excretion approximately equals endogenous production (Figure 2) [47,49]. This has been demonstrated in both rats and pigs, where more than 95% of an orally administered dosage of homoarginine was recovered unmetabolized in the urine [50]. The most interesting findings come from a comparison of data in healthy donors before and at approximately six weeks after living kidney donation [51,52]. At six weeks after donation, the decline in kidney function both according to the true glomerular filtration rate (GFR) and to the estimated GFR was on average 37% (Table 1). While this is acknowledged to be lower than the 50% decline that might be anticipated, this was because of the known phenomenon of compensatory hypertrophy of the remaining kidney [53]. Meanwhile, there is a significant 12% decline in plasma homoarginine concentrations, and a 25% decline in urinary homoarginine excretion, both consistent with decreased production of homoarginine by the remaining kidney. Of note, the percentage decrease in homoarginine production is approximately two-thirds of the percentage decrease in true GFR and the estimated GFR, which strongly suggests that the observed decline in kidney function and decline in AGAT activity go hand in hand. This was observed in the absence of being controlled on a creatine-free diet, because our healthy kidney donors were not on such a diet. Of further note, is that the decline in plasma guanidinoacetate concentration was greater than the decline in plasma homoarginine concentration. This was likely because of stimulated endogenous synthesis of creatine, which is not inconceivable at six weeks after the donation of a kidney for transplantation. These combined results suggest that the metabolic capacity of AGAT is highly dependent on functional kidney mass and that decline in renal AGAT activity is at most partially compensated for by extra-renal AGAT activity, such as in the pancreas and brain. The suggestion that the metabolic capacity of AGAT in the kidney declines with functional kidney mass is also consistent with the notion that AGAT resides in the kidney tubules and that tubular atrophy is a hallmark of progressive CKD [54,55]. Together, these observations strongly suggest that the capacity for synthesis of guanidinoacetate by kidneys progressively decreases with increasing stages of CKD, to become absent or virtually absent in dialysis patients. However, even if kidneys would only contribute 20% to total endogenous guanidinoacetate synthesis, incomplete compensation for this loss of function by extra-renal sites of synthesis would pose patients at risk of negative creatine balance if exposed to a low-protein diet, particularly if that low-protein diet would be mainly plant-based [27]. Although this phenomenon might play a role over the continuum of stages of CKD, it should be present in patients that soon will start with dialysis and the most extreme situation should be present in patients on dialysis. In patients entering dialysis, this should be demonstrable by a particularly high prevalence of sarcopenia in this group. 

## 4. Sarcopenia in CKD

Reported prevalence rates of sarcopenia depend on the applied methods and criteria, with the use of criteria formulated by the European Working Group on Sarcopenia in Old People (EWGSOP) resulting in much lower reported prevalence rates than with use of the criteria formulated by the Foundation for the National Institutes of Health (FINH) Sarcopenia project [56]. The latter criteria again result in much lower prevalence rates than prevalence rates with the use of criteria based on the assessment of appendicular lean mass index assessed via dual-energy X-ray absorptiometry [56]. So it is difficult to compare prevalence rates between studies, but within studies, there is a gradual increase in the prevalence rates of muscle weakness, low muscle mass, and sarcopenia, with increasing stages of non-dialysis-dependent CKD [19,56,57,58]. For example, in the NHANES III population, in which sarcopenia was assessed by means of measured bio-electrical impedance analysis (BIA), the prevalence of sarcopenia ranged from 26.6% in subjects with an estimated glomerular filtration rate (eGFR) of ≥90 mL/min/1.73 m^2^ to 60.1% in non-dialysis-dependent patients with an eGFR of <60 mL/min/1.73 m^2^ [59]. In a Brazilian study, in which sarcopenia was assessed by means of dual-energy X-ray absorptiometry, the prevalence of sarcopenia ranged from 34.5% in patients with an eGFR between 45 and 90 mL/min/1.73 m^2^ to 65.5% in patients with an eGFR <45 mL/min/1.73 m^2^ [56]. Because creatine in muscles is continuously converted to creatinine at a rate of approximately 1.6–1.7% of its mass per day [27], 24 h urinary creatinine excretion is considered a reliable measure of muscle mass, even in patients with advanced renal failure, in children and adolescents, elderly people, and patients with wasting conditions [60,61,62,63,64]. Using 24 h urinary excretion of creatinine as marker of muscle mass, we recently found that sarcopenia—as defined by low creatinine excretion—was prevalent in 38% of the patients with advanced CKD, with prevalence increasing with the severity of CKD and the risk for low creatinine excretion being 25.8 times higher in patients just before the start of dialysis, as compared to patients with less advanced CKD [65]. Studies that used other methods to measure muscle mass found a similar association of impaired kidney function with low muscle mass [59,66,67,68]. Importantly, this low muscle mass is not without consequences. One of the most compelling observations is that low muscle mass, as evidenced by low 24 h urinary creatinine excretion, is a very strong independent predictor of mortality in patients with CKD [68]. The same is true for patients with dialysis-dependent CKD, in which low 24 h urinary creatinine excretion just prior to dialysis has been shown to be a very strong independent predictor of premature mortality after the start of dialysis [60]. Less evident, but from the patient perspective, possibly even more compelling, is that CKD—particularly in its advanced stages—is also associated with fatigue, poor cognition, depression and low quality of life [19,57,65,69,70,71,72]. Interestingly, these symptoms are similar to those observed in patients with a genetic deficiency of AGAT [73]. Importantly, in this genetic condition, the muscle symptoms are entirely reversible by creatine supplementation [73]. If creatine supplementation starts at a young age, the occurrence of cognitive dysfunction is reversible and even preventable [74,75,76]. 

## 5. Most Susceptible Patient Group

Many complications in CKD begin to occur once the GFR falls below 60 mL/min/1.73 m^2^ (CKD stage 3) [77,78,79]. It is conceivable that the same applies for creatine deficiency. Susceptibility for development of creatine deficiency would further increase as CKD progresses to stage 4 and 5, with the highest risk after initiating dialysis. In these patients, the contribution of the kidneys to endogenous guanidinoacetate synthesis will be absent or virtually absent and losses of guanidinoacetate and creatine into dialysate may be high. Actually, in these patients, higher protein intake, higher energy intake and multivitamin supplementation are recommended to compensate for the relatively excessive losses of small molecular substances like amino acids, peptides, glucose, vitamins and micronutrients into the dialysate during dialysis [20,21,22,23,24]. Because creatine and guanidinoacetate are small molecular weight substances, it is likely that they are also subject to significant losses during dialysis, but their supplementation is currently not included in recommendations. Rather, currently prevailing recommendations towards a preference for plant-based foods to allow for maintenance of acid-base and phosphate homeostasis [20], may be adverse if one would consider the maintenance of muscle mass and cognitive function an important goal.

## 6. Hypothesis

**Figure 3:**
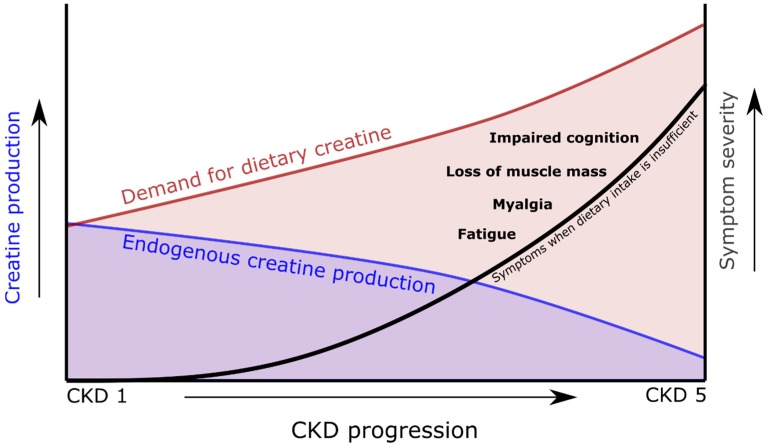
Schematic overview indicating the increasing demand for dietary creatine as endogenous creatine production falls during chronic kidney disease (CKD) progression.

We hypothesize that with increasing stages of CKD, creatine coming from meat and dairy in food increasingly becomes an essential nutrient (Figure 3). This phenomenon will be most pronouncedly present in patients with dialysis-dependent CKD, because of the combination of low endogenous production of creatine and the unopposed losses of creatine and its precursor guanidinoacetate into the dialysate. With an increasing focus on a plant-based intake, it is likely that these increased demands for dietary creatine are not sufficiently met. This would result in a creatine deficiency, with important contributions to sarcopenia, fatigue, impaired quality of life, impaired cognition, and premature mortality seen in CKD. It is also important to consider that levels of phosphocreatine concentrations in muscle have been reported to be as high as over 30 mmol/L [26]. So, if a patient with dialysis-dependent CKD would swing back and forth between creatine sufficiency and insufficiency, or even be in a continuous state of creatine insufficiency, periods of muscle catabolism due to creatine insufficiency could possibly: Explain episodes of spurious hyperphosphatemia, which frequently occur; lead to false suspicion of non-adherence to diet or phosphate binder regimens; and most importantly, impair the quality of life of patients [80,81,82].

## 7. Effects on Creatine Supplementation

The International Society of Sports Nutrition has stated that creatine is the most effective food supplement available for improving high-intensity performance and increasing lean muscle mass [83]. Though creatine supplementation has sparsely been studied in CKD, there are extensive data on creatine supplementation in healthy young individuals, athletes and elderly. Several meta-analyses performed in young adults concluded that creatine supplementation, with or without resistance training not only increased lean body mass, but also strength and performance during short-term intense exercise [84,85,86]. In addition, a meta-analysis in older men concluded that creatine supplementation in combination with resistance training was more effective than resistance training without creatine supplementation in increasing body weight, fat-free mass and muscle strength [87]. Several studies also found an increase in fat-free mass in older adults using creatine without resistance training [88,89]. These same studies also found a beneficial effect on daily tasks, such as a reduction in the time to complete sit-stand and tandem gait tests. One of the few studies performed in hemodialysis patients, investigated the effect of creatine supplementation on muscle cramps in 10 patients and found a 60% reduction of muscle cramps while on creatine supplementation. After a wash-out period, these muscle cramps returned [90]. While the effects of creatine supplementation on quality of life and cognition have been studied less, the available results are largely positive. In patients with Parkinson’s disease, creatine supplementation improved patient mood [91] and in a study in elderly, creatine supplementation resulted in significant improvements in cognitive tasks [92]. A review of randomized controlled trials in healthy individuals concluded that creatine supplementation seems to improve short-term memory and intelligence/reasoning, while the effects on other cognitive domains remain unclear [93]. An extensive overview of the clinical trials performed with creatine supplementation is given by Gualano et al. [94].

## 8. Safety of Creatine Supplementation

As the amount of randomized clinical trials using creatine supplementation is growing, so is the support for its safety. Studies both in athletes and in the general populations have shown that creatine supplementation, varying from a few days to five years, does not lead to adverse changes in markers of clinical health, including renal function [95,96,97,98,99,100]. In addition, recent research has shown that creatine supplementation, unlike what has previously been thought, does not lead to the formation of carcinogenic heterocyclic amines [101]. However, it should be noted that little is known regarding the safety of creatine supplementation in patients with CKD. Despite the biological plausibility for creatine deficiency being present in patients with CKD, particularly in patients with dialysis-dependent CKD, there are too few data available to position creatine deficiency playing a role in symptoms and problems in patients with CKD currently as more than a hypothesis. More research should be done to support this hypothesis and if such data become available, randomized clinical trials should be performed to demonstrate safety and benefits before creatine supplementation can actually be recommended in this setting.

Another point worth noting, is that the influence creatine supplementation might have on the assessment of renal function. Serum creatinine has become the most used biomarker for assessing renal function. As creatine is non-enzymatically converted to creatinine, creatine supplementation will invariably increase serum creatinine, which may falsely suggest renal function deterioration if injudiciously interpreted. Indeed, several case studies have been reported where creatine supplementation led to misdiagnosis of renal failure [102,103,104]. However, a clinical trial consisting of a 12-week creatine supplementation showed a significant increase in serum creatinine, while another marker for renal function, cystatin C, remained unchanged [99]. Other studies found no change in either serum creatinine or other biomarkers of renal function after creatine supplementation [97,105,106]. It should also be noted that the degree to which creatine supplementation increases serum creatinine may depend on whether creatine is supplemented as creatine monohydrate or creatine ethyl ester. Creatine ethyl ester has a higher solubility and is claimed to have better bioavailability [102,107]. Creatine ethyl ester can be degraded to creatinine in the gastrointestinal tract and has been shown to increase serum creatinine more than creatine monohydrate [102,107]. Either way, clinicians should be aware of the potential influence that creatine supplementation may have on the eGFR based on serum creatinine, while there is no actual change in renal function. Other biomarkers for renal function, such as cystatin C, could be used to circumvent this. 

## 9. Limitations

Several limitations need to be addressed. First, we hypothesize that creatine synthesis decreases with increasing renal function impairment and may, therefore, increasingly become an essential nutrient. It does, however, remain unknown to what extent the diet can meet this increasing demand. Even if plant-based foods are increasingly recommended, most official guidelines do not yet state a preference for plant-based protein intake over animal protein intake. In fact, K/DOQI guidelines recommend that at least 50% of the dietary protein intake for patients treated with maintenance hemodialysis and chronic peritoneal dialysis should be of high biological value (i.e., likely of animal source) [9,13,14]. In addition, patient adherence to diet is often poor in patients on CKD, so it is possible that creatine demands are still met [108]. Therefore, studies are warranted to assess whether creatine intake in CKD patients meets the increased creatine demands or not.

Secondarily, no causal links have been established between creatine levels and CKD symptoms, or sarcopenia, muscle weakness, fatigue or impaired cognition. As these symptoms are multifaceted, other factors likely contribute. These other factors include low physical inactivity, inflammation, metabolic acidosis, activation of the ubiquitin–proteasome system and defective insulin signaling, which have all been implicated in the loss of muscle and weakness related to CKD [19,109,110,111,112].

## 10. Conclusions

Many CKD patients suffer from fatigability, muscle weakness, muscle wasting, and sarcopenia. These symptoms are similar to those in patients with a genetic deficiency in the enzyme required for creatine synthesis. Creatine is an essential contributor in cellular energy homeostasis, yet on a daily basis, 1.6–1.7% of the total creatine pool is degraded. To accommodate for these losses, a combination of dietary creatine intake and endogenous creatine synthesis is required. Endogenous creatine synthesis involves two enzymatic steps, of which the first step is a metabolic function of the kidney, facilitated by the enzyme AGAT. Recent findings strongly suggest that the capacity of renal AGAT, and thus endogenous creatine production, progressively decreases with increasing stages of CKD, to become absent or virtually absent in dialysis patients. We hypothesize that with an increasing stage of CKD, creatine coming from meat and dairy in food increasingly becomes an essential nutrient. This phenomenon will be most pronouncedly present in patients with dialysis-dependent CKD, because of the combination of the lowest endogenous production of creatine and the unopposed losses of creatine into the dialysate. With an increasing focus on a plant-based intake, it is likely that these increased demands for dietary creatine are not sufficiently met. The result being a creatine deficiency, with important contributions to the sarcopenia, fatigue, impaired quality of life, impaired cognition, spurious hyperphosphatemia, and premature mortality seen in CKD. Further studies are necessary to investigate the potential of increasing dietary creatine or creatine supplements in patients with dialysis-dependent CKD, and—if shown to be true—also in pre-dialysis patients with CKD stages 3–5.

## Figures and Tables

**Table 1:** Effect of unilateral nephrectomy on biochemical parameters in 127 healthy kidney donors. Study design and methods are described previously [51,52].

Variable	*N*	Before Donation	After Donation	Absolute Difference	Change in Mean (%)	*p*-Value
Urinary homoarginine excretion (µmol/24 h)	127	4.0 ± 4.4	3.0 ± 2.3	1.1 ± 3.4	−25	0.001
Urinary urea excretion (mmol/24 h)	125	420 ± 127	394 ± 111	26 ± 140	−6	0.04
Urinary sodium excretion (mmol/24 h)	125	203 ± 72	176 ± 65	27 ± 82	−13	<0.001
Plasma homoarginine (µmol/L)	125	1.7 ± 0.6	1.5 ± 0.5	0.2 ± 0.4	−12	<0.001
Plasma guanidinoacetate (µmol/L)	127	2.9 ± 1.1	2.3 ± 0.5	0.6 ± 1.0	−23	<0.001
Serum creatinine (µmol/L)	127	73 ± 12	107 ± 21	−35 ± 11	+47	<0.001
mGFR (mL/min)	127	118 ± 24	74 ± 14	43 ± 14	−37	<0.001
eGFR (mL/min/1.73 m^2^)	127	95 ± 16	60 ± 12	34 ± 10	−37	<0.001

Abbreviations: eGFR: estimated glomerular filtration rate. mGFR: measured glomerular filtration rate.
